# Revealing Antidepressant Mechanisms of Baicalin in Hypothalamus Through Systems Approaches in Corticosterone- Induced Depressed Mice

**DOI:** 10.3389/fnins.2019.00834

**Published:** 2019-08-08

**Authors:** Kuo Zhang, Meiyao He, Fan Wang, Haotian Zhang, Yuting Li, Jingyu Yang, Chunfu Wu

**Affiliations:** Department of Pharmacology, Shenyang Pharmaceutical University, Shenyang, China

**Keywords:** baicalin, hypothalamus, proteomics, depression, glucocorticoid receptor

## Abstract

Baicalin, the main active flavonoid constituent of *Scutellaria baicalensis* Georgi, has been reported to exert antidepressant effects. Hypothalamic-pituitary-adrenal (HPA) axis plays important roles in depression. However, antidepressant effect and mechanism of baicalin on HPA axis in hypothalamus are still unknown. In present study, we find baicalin significantly attenuates the increase of immobility time in tail suspension and forced swimming, improves the decrease of spending time in open arms, and restores the aberrant negative feedback of HPA axis in chronic corticosterone (CORT)-induced depressed mice. Moreover, proteomics finds 370 differentially expressed proteins after baicalin treatment, including 114 up-regulation and 256 down-regulation in hypothalamus. Systems biology analysis indicates the functions of differentially expressed proteins focus on phosphoserine binding and phosphorylation, especially participate in GR signaling pathway. Finally, our findings demonstrate that baicalin normalizes hypothalamic GR nuclear translocation via reducing GR phosphorylation to remodel negative feedback of HPA axis in CORT-induced mice.

## Introduction

Depression is the kind of affective disorder, which seriously threatens human health and brings heavy social burdens (McEwen et al., 2015). Most antidepressant drugs are developed according to the phenomenon which deficient monoamine neurotransmitters are discovered in depressive patients (Boku et al., 2018). However, patients are usually unsatisfied with the therapeutic effects due to the delayed actions and side effects (Duman and Aghajanian, 2012). Many studies show that the abnormal HPA axis participates in depression (Anacker et al., 2011b). Especially, hypothalamus has important modulatory function in brain, which controls the activity of hypothalamic-pituitary-adrenal (HPA) axis and responds to the stress (Myers et al., 2014). However, the role of hypothalamus in abnormal HPA axis and the molecular mechanisms of antidepressant drug in hypothalamus have not been definitely illuminated. Proteomics is new-style development of biological systems, which possessed the powerful capacity to analyze proteins by high throughput (Frantzi et al., 2019). Moreover, proteomics is also the powerful tool to explore the complex system of disease and the therapeutic target of new drug (Jiang et al., 2019).

Baicalin, the main active flavonoid constituent of *Scutellaria baicalensis* Georgi, which has reported to exert multiple pharmacological actions, including anti-inflammatory, anti-tumor, anti-ischemia (Li-Weber, 2009; Hou et al., 2012; Luan et al., 2019). Recent studies show baicalin has definite antidepressant-like activity which can improve olfactory functions by inhibiting APPL2-mediated glucocorticoid receptor (GR) hyperactivity (Gao et al., 2018). However, the effects of baicalin on HPA axis are still unknown. The definite molecular targets of baicalin on HPA axis in hypothalamus need to be further investigated.

In the present study, our results demonstrate that baicalin remarkably improves chronic corticosterone (CORT)-induced various depression-like behaviors and restores the negative feedback of HPA axis. Proteomics and systems biology indicate that the molecular mechanisms of baicalin on negative feedback of HPA axis involve normalizing GR nuclear translocation via regulating GR phosphorylation in hypothalamus. Our findings provide the new perspective on molecular targets of baicalin and will facilitate its application in clinic.

## Materials and Methods

### Ethics Statement

Adult C57BL/6 male were supplied by the Experimental Animal Center of Shenyang Pharmaceutical University [License number: SYXK (Liao), 2014-0004]. This study was carried out in accordance with the principles of National Institutes of Health Guide for the Care and Use of Laboratory Animals (Publication No. 85-23). All efforts were made to minimize suffering. The protocol was approved by the local ethic committee of Shenyang Pharmaceutical University.

### Animals, Drugs, and Biochemical Reagents

Adult 8 week old C57BL/6 male mice weighing 18–22 g are supplied by the Experimental Animal Centre of Shenyang Pharmaceutical University. Animals are fed in standardized environment of 12 h light and dark cycle, with room temperature at 22 ± 2°C. Mice are given free access to food and water, adapted for 7 days before experiment.

Baicalin (purity >99%) is purchased Nanjing Zelang Medical Technology Company Limited in China. The mouse anti-GR (ab2768), anti-phospho-GR (S203, ab195703), anti-phospho-GR (S226, ab195789) are purchased from Abcam. The anti-phospho-GR (S211, 4161) is purchased from CST. The anti-β-actin (sc-47778) is purchased from Santa Cruz. The rabbit anti-Lamin B1 (12987-1-AP) is purchased from Proteintech.

### Group and Drug Treatment

**FIGURE 1:**
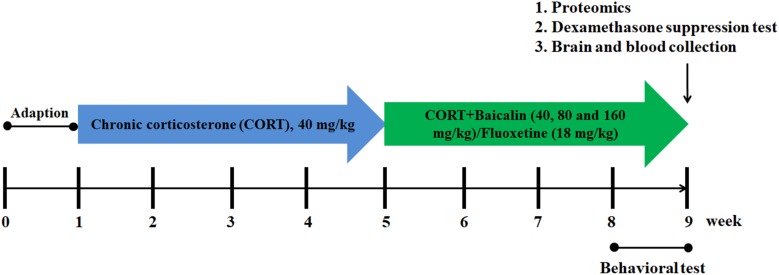
Schematic representation of the experimental procedure.

A total of 90 mice are randomly assigned to six groups (*n* = 15/group), including control group, CORT group, baicalin (40, 80, and 160 mg/kg) group, and fluoxetine (18 mg/kg) group. Among them, eight mice randomly selected from each group are used in behavioral testing. Four mice randomly selected from each group are used in western blot analysis. Three mice randomly selected from each group are used in proteomic analysis. The mice of western blot analysis and proteomic analysis are not used in behavioral testing to avoid behavior influence. The doses of baicalin in this study according to our previous study (Zhang et al., 2016). The procedure of CORT administration is performed as previously described (Wu et al., 2013). In brief, mice are injected subcutaneously with CORT (40 mg/kg, Tokyo Chemical Industry) which is dissolved in saline (0.45% Hydroxypropyl-β-Cyclodextrin, Sigma) between 8:00 am and 10:00 am for 8 weeks. Pharmacological treatment started in the 4th week after the beginning of the CORT protocol. Baicalin and fluoxetine are administrated by gastric gavages 30 min prior to the corticosterone injection until the end of the experiment. Detailed experimental procedure is showed in Figure 1.

### Tail Suspension Test

Tail suspension test is executed according to previous study (Steru et al., 1985). The mice are respectively, pasted on the suspension instrument at 1 cm from the tip of the tail with the medical adhesive strip. The behavior of mice is recorded by the high-definition camera for 6 min. In brief, the mice are firstly adaptively suspended for 2 min, and then accumulated the immobility time for remaining 4 min. The video is analyzed by video traceable system (Ethovision Vision-XT 8.0).

### Forced Swimming Test

Forced swimming test is executed according to previous study (Porsolt et al., 1978). The mice are respectively, placed in a plexiglass cylinder (height 40 cm, diameter 12 cm) for 6 min, which is filled at the depth of 10 cm with 23 ± 2°C water. The behavior of mice is recorded and observed by the high-definition camera. In brief, the mice are firstly adapted for 2 min in the water, and then accumulated the immobility time for remaining 4 min. The video is analyzed by video traceable system (Ethovision Vision-XT 8.0).

### Elevated Plus Maze

Elevated plus maze is executed according to previous study with some modifications (Nollet et al., 2012). The mice are placed in the center of the maze, and their heads are orientated to the open arm, and the accumulated time of the mice entered in the arms is recorded for 5 min. The behavior of mice is recorded and observed by the high-definition camera. The video is analyzed by video traceable system (Ethovision Vision-XT 8.0).

### Dexamethasone Suppression Test

Eight mice randomly selected from each group are used in this test. In brief, mice are intraperitoneally injected with dexamethasone (0.1 mg/kg in 0.9% NaCl, *n* = 4) or saline (0.9% NaCl, *n* = 4). Then, mice are suffered by the stressor after 30 min under dexamethasone or saline treatment. Finally, mice are anesthetized and serum are collected for serum corticosterone analyses after 120 min under dexamethasone or saline treatment. The rate of DEX-induced CORT suppression was calculated as a ratio of the amount of the level of corticosterone under dexamethasone treatment to the level of corticosterone under saline treatment: the rate of DEX-induced CORT suppression (%) = the level of corticosterone under dexamethasone treatment/the level of corticosterone under saline treatment.

### Measurement of Serum Corticosterone

Blood samples are separated by refrigerated centrifuge (4000 rpm, 5 min). Serum corticosterone is measured by ELISA kits according to the operating guide.

### Western Blot Analysis

Four mice randomly selected from each group are used in this test. The hypothalamus is homogenized in cold RIPA buffer containing 1 mM PMSF, 1 mM NaF and 1 mM Na_3_VO_4_ for 30 min. The samples are centrifuged at 12000 rpm for 20 min at 4°C. The concentration of protein is detected by bicinchoninic acid method. Then, denatured proteins (20–25 ug) are separated by 12% SDS-PAGE and transferred to PVDF membranes (Millipore). The membranes are steeped with 5% non-fat milk for 1 h, then incubated with primary antibody at 4°C overnight and appropriate secondary antibody at room temperature for 60 min. Finally, membranes are visualized and analyzed by Image J software.

### Proteomics and Systems Biology Analysis

In present study, we tested three doses of baicalin (40, 80, and 160 mg/kg) in several behavioral testing and confirmed that 160 mg/kg was the best effective dose of baicalin on depression. As a consequence, we chose the best effective dose 160 mg/kg of baicalin to analyze quantitative proteomics by iTRAQ. Three mice randomly selected from each group are used in this test. At the end of experiment, 1 h after baicalin/CORT administration, mice were euthanized and sacrificed, then hypothalamus was quickly isolate. The samples are homogenized by lysis buffer and centrifuged. The supernatant is filtered and digested. iTRAQ reagent (AB SCIEX) is applied to label the peptide mixture. The peptide mixture is fractionated by SCX chromatography. LC-MS/MS analysis is operated by Q Exactive mass spectrometer. Detailed conditions are executed according to our previous study. Systems biology analysis contains gene ontology (GO) annotation and enrichment analysis, clustering analysis and protein-protein interaction analysis. In GO annotation, the functions of proteins are analyzed by cellular component, molecular function, and biological process. In clustering analysis, hierarchical clustering is carried out by euclidean distance algorithm and average linkage clustering algorithm. In protein-protein interaction analysis, the database of STRING is used. The importance of the node can be evaluated by degree of node in protein-protein interaction network.

### Statistical Analyses

All data are analyzed by SPSS 22.0 and expressed as the mean ± SEM. Data are analyzed by one-way ANOVA followed by *post hoc* Fisher’s LSD test. *P* < 0.05 is considered to be statistically significant.

## Results

### Effects of Baicalin on Depression-Like Behaviors in CORT-Induced Mice

**FIGURE 2:**
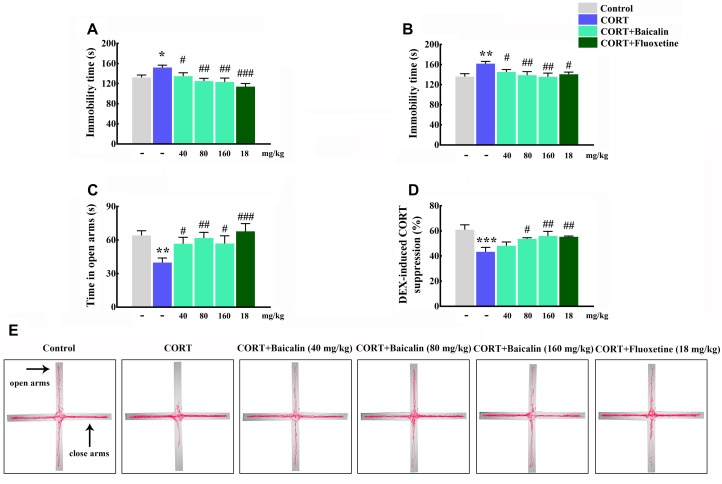
Effects of baicalin on tail suspension test **(A)**, forced swimming test **(B)**, elevated plus maze **(C)**, and DEX-induced corticosterone suppression **(D)** in CORT-induced mice. Representative movement tracks in the elevated plus maze are showed in **(E)**. Data are expressed as means ± SEM (*n* = 8–12 mice/group). ^*^P < 0.05, ^∗∗^*P* < 0.01, and ^∗∗∗^*P* < 0.001 vs. control. ^#^*P* < 0.05, ^##^*P* < 0.01, and ^###^*P* < 0.001 vs. CORT model.

It had been demonstrated that chronic CORT could induce multiple anxiety/depression-like behaviors in mice. Therefore, we first verified the establish of CORT-induced depression model, and then assessed the protective effects of baicalin in CORT-induced mice. The results showed that the immobility time in tail suspension test and forced swimming test was increased by chronic CORT (*P* = 0.024, *P* = 0.002), while baicalin (tail suspension test, *P* = 0.049, *P* = 0.003 and *P* = 0.0011; forced swimming test, *P* = 0.046, *P* = 0.006, and *P* = 0.002) and fluoxetine treatment could recover this enhancement (Figures 2A,B). In elevated plus maze test (Figure 2E), the spending time of mice in open arms was decreased by chronic CORT (*P* = 0.003), but application of baicalin (*P* = 0.037, *P* = 0.007, and *P* = 0.035) and fluoxetine could recover this decline (Figure 2C). These data indicate that baicalin has definite antidepressant effect in CORT-induced mice.

### Effects of Baicalin on Negative Feedback of HPA Axis in CORT-Induced Mice

Next, in order to explore potential mechanism of baicalin, the negative feedback of HPA axis was assessed by DEX suppression test. The results showed that the ratio of DEX-induced serum corticosterone suppression was decreased by chronic CORT (Figure 2D, *P* = 0.0004). On the contrary, the reducing of DEX-induced serum CORT suppression could be reversed by baicalin (*P* = 0.023, *P* = 0.007) and fluoxetine treatment (Figure 2D). These data suggest that baicalin has definite regulatory effect on the abnormal negative feedback of HPA axis.

### Proteomics and Systems Biology Uncover Molecular Mechanisms of Baicalin in Hypothalamus in CORT-Induced Mice

**FIGURE 3:**
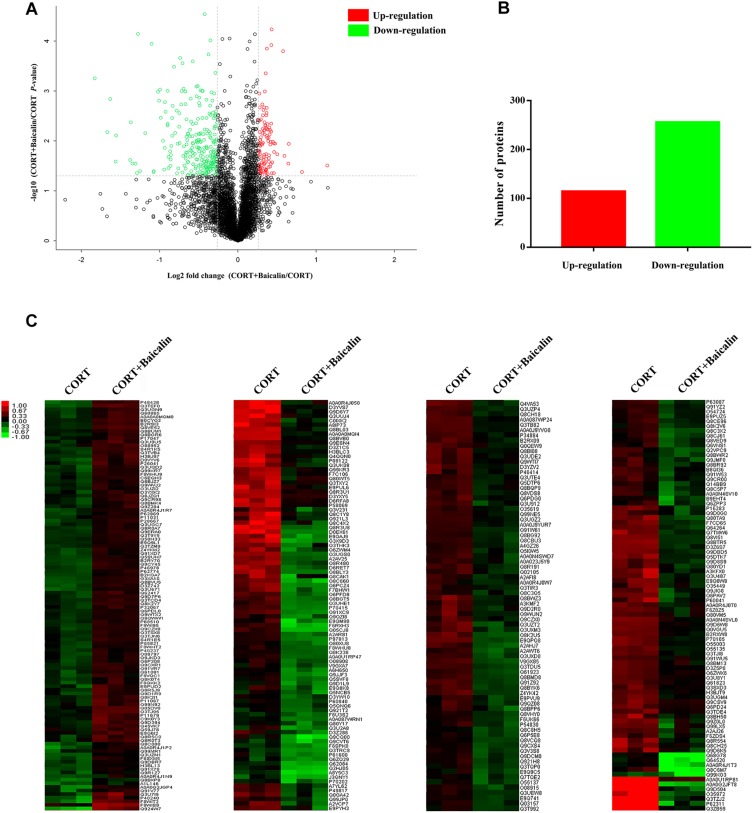
Volcano plot **(A)**, quantitative analysis **(B)**, and hierarchical clustering **(C)** of differentially expressed proteins after baicalin treatment in CORT-induced mice. The log2-transformed value of cluster analysis was showed by red-green color scale **(C)**. Red meant high expression and green meant low expression.

In order to reveal the precise molecular mechanisms of baicalin on negative feedback of HPA axis, isobaric tags for relative and absolute quantification (iTRAQ) quantitative proteomics was used to assess the hypothalamic differentially expressed proteins after baicalin treatment in CORT-induced mice. A total of 4776 proteins were confirmed by high reliability at 1% false discovery rate (Figure 3A). Then, further analysis found 370 differentially expressed proteins after baicalin treatment, including 114 up-regulation and 256 down-regulation (Figure 3B). The detailed information of differentially expressed proteins was showed in Supplementary Table S1. Then, to verify the veracity of differentially expressed proteins, hierarchical clustering analysis was used. The result showed that differentially expressed proteins after baicalin and chronic CORT treatment were separated into two obvious distinguishing branches (Figure 3C). It is definitely indicated that these proteins have distinct expression between baicalin and chronic CORT group.

**FIGURE 4:**
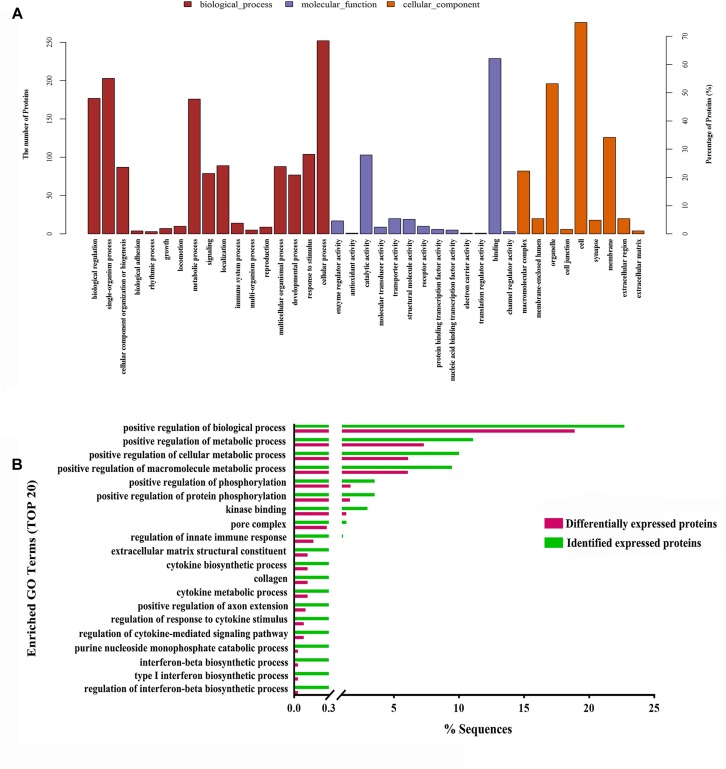
GO functional annotations **(A)** and GO enrichment analysis **(B)** of differentially expressed proteins after baicalin treatment in CORT-induced mice.

**FIGURE 5:**
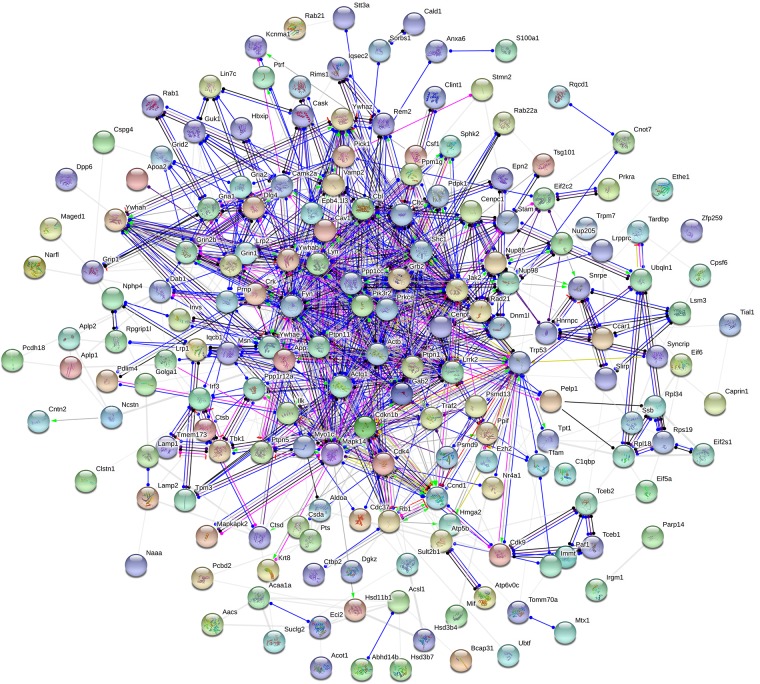
Protein-protein interaction network of differentially expressed proteins after baicalin treatment in CORT-induced mice.

Moreover, to further uncover the function of differentially expressed proteins, differentially expressed proteins were assessed by GO functional annotations and enrichment analysis. In biological process, differentially expressed proteins mainly involved cellular process, single-organism process, and metabolic process (Figure 4A). In molecular function, differentially expressed proteins mainly involved binding and catalytic activity (Figure 4A). In cellular component, differentially expressed proteins mainly involved cell, organelle and membrane (Figure 4A). Then, GO enrichment showed that positive regulation of biological process, positive regulation of metabolic process, positive regulation of phosphorylation, and nuclear pore complex might be the chief functions of differentially expressed proteins (Figure 4B). Next, protein-protein interaction analysis was used to predict potential molecular targets of baicalin by STRING (Figure 5). According to the degree of nodes, the name, molecular function and biological process of high degree nodes were showed in Supplementary Table S2. Among them, a large number of molecular function and biological process indicated that GR signaling pathway, especially GR binding, phosphoserine binding and protein phosphorylation, and might be the molecular targets of baicalin on negative feedback of HPA axis in hypothalamus.

### Effects of Baicalin on Hypothalamic GR Nuclear Translocation and Phosphorylation in CORT-Induced Mice

**FIGURE 6:**
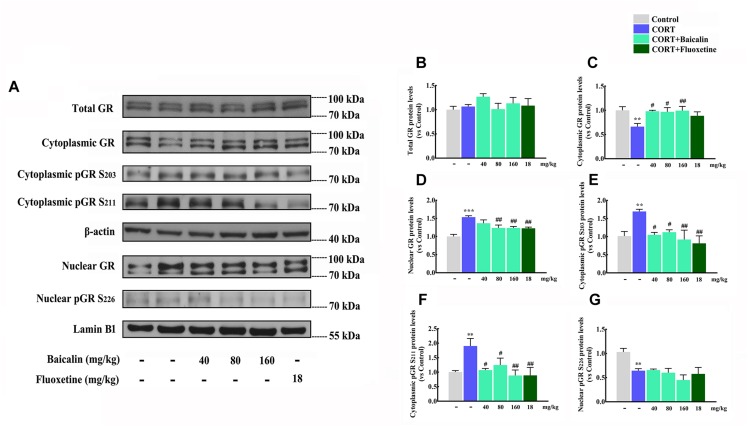
Effects of baicalin on GR nuclear translocation and phosphorylation in CORT-induced mice. Representative western blots of GR and phosphorylated GR expression are showed **(A)**. Quantification analyze of total GR **(B)**, cytoplasmic GR **(C)**, nuclear GR **(D)**, cytoplasmic pGR S203 **(E)**, cytoplasmic pGR S211 **(F)**, and nuclear pGR S226 **(G)**. Data are expressed as means ± SEM (*n* = 3–5 mice/group). ^∗∗^*P* < 0.01 and ^∗∗∗^*P* < 0.001 vs. vehicle. ^#^*P* < 0.05, ^##^*P* < 0.01 vs. CORT model.

According to the results of proteomics and systems biology, we focus on the level of GR, GR nuclear translocation and phosphorylation in hypothalamus (Figure 6A), which might be antidepressant molecular targets of baicalin. Our results showed that the level of total GR in hypothalamus had no change under chronic CORT, baicalin or fluoxetine treatment (Figure 6B, *P* = 0.658). It was noteworthy that the level of GR in cytoplasm was decreased (*P* = 0.007) and the level of GR in nucleus was increased by chronic CORT (Figure 6C, *P* < 0.001). The decline of GR in cytoplasm could be improved by baicalin (*P* = 0.0101, *P* = 0.012, and *P* = 0.008), but fluoxetine had no effect on this decline (Figure 6C). In addition, the enhancement of GR in nucleus could be decreased by both baicalin (*P* = 0.002, *P* = 0.002) and fluoxetine treatment (Figure 6D). Moreover, the phosphorylation status of the crucial serine residues was detected, which could play the important role in regulating GR nuclear translocation. The level of pSer203 and pSer211 in cytoplasm was increased by chronic CORT (*P* = 0.0079, *P* = 0.0081), and this enhancement could be decreased by baicalin (pSer203, *P* = 0.011, *P* = 0.02, and *P* = 0.004; pSer211, *P* = 0.012, *P* = 0.039, and *P* = 0.004) and fluoxetine treatment (Figures 6E,F). The pSer226 level in nucleus was decreased by chronic CORT (*P* = 0.008), but no effects were observed by baicalin and fluoxetine treatment (Figure 6G). Above data indicate that baicalin can recover abnormal GR nuclear translocation by regulating GR phosphorylation.

## Discussion

Depression is a common affective disorder, which people persistently feel loneliness and sadness. Although depression seriously threatens human health, the pathophysiological mechanism of depression is still unknown (Blier, 2016). Clinical studies find that major depression disorders usually exhibit high level of CORT in serum, whereas antidepressant drugs decrease CORT level, and improve depressive symptom (Pariante and Lightman, 2008). Moreover, previous studies also demonstrate that chronic CORT induces depression-like behaviors in mice (David et al., 2009; Wu et al., 2013). Baicalin, one of predominant flavonoid compounds in Radix Scutellariae, has showed definite antidepressant effects. For example, baicalin exerted antidepressant effects which improved olfactory functions by inhibiting APPL2-mediated GR hyperactivity in olfactory bulb (Gao et al., 2018). Our previous study showed that baicalin also promoted hippocampal neurogenesis via SGK1 and FKBP5-mediated GR phosphorylation in hippocampus (Zhang et al., 2016). However, the antidepressant effects and mechanisms of baicalin in hypothalamus are still unknown and need to be further investigated. Therefore, in present study, we establish the chronic CORT-induced mouse model of depression to assess the antidepressant effect and mechanism of baicalin. Tail suspension test and forced swimming test are two classic behavioral despair model, and the immobility time of mice will be decreased if drugs have definite antidepressant effect. On the other hand, elevated plus maze is a contradictory conflict test: the drive to new environment and the fear to enter high and dangling open arms, and anxiolytic drugs can increase the spending time in open arms. The same as previous study (David et al., 2009), our study finds that chronic CORT significantly increases the immobility time in tail suspension test and forced swimming test and decreases the spending time in open arms, which indicates that the model of depression has been successfully established and make sure chronic CORT indeed induces anxiety/depression-like behaviors. After baicalin treatment, above anxiety/depression-like behaviors are significantly improved. These data demonstrate that baicalin has definite antidepressant-like activity in CORT-induced mice.

The HPA axis is important neuroendocrine system, which regulates stress response, emotion, digestion and immune system (Pariante and Lightman, 2008). Chronic stress persistently activates HPA axis and leads to long period high level of CORT. More importantly, hyperactivity of the HPA axis usually is found in major depression disorders (Tsigos and Chrousos, 2002). Above phenomenon indicates the negative feedback of HPA axis is damaged in depression. Moreover, some studies show unpredictable chronic mild stress destroys negative feedback of HPA axis, and antidepressant drugs improve this damage (Nollet et al., 2012). Based on the importance of HPA axis, we evaluate the effect of baicalin on negative feedback of HPA axis in CORT-induced mice by dexamethasone suppression test. Dexamethasone is an artificially synthesized powerful glucocorticoid, which can suppress the release of CORT (Anacker et al., 2011a). Then, the ratio of DEX-induced serum CORT suppression is significantly decreased by chronic CORT, which indicates that chronic CORT destroys negative feedback of HPA axis. The same as clinic treatment, fluoxetine can restore the negative feedback of HPA axis in CORT mice. Interestingly, baicalin normalizes DEX-induced serum CORT suppression rate. These data demonstrate that baicalin exerts antidepressant effect by restoring negative feedback of HPA axis, but accurate regulatory mechanisms and targets need to be illuminated.

Aim to dissect the regulatory mechanisms and targets of baicalin on negative feedback of HPA axis, proteomics and systems biology to are applied in this study. Proteomics is a scientific and systematic approach for clarifying the interrelation of proteins, which has been widely applied to search targets of drugs (Frantzi et al., 2019). In our study, 370 differentially expressed proteins (114 up-regulation and 256 down-regulation) in hypothalamus are found after baicalin treatment by iTRAQ quantitative proteomics. Among them, some differentially expressed proteins have been reported to involve in anxiety and depression-like behavior, such as Nrxn2 and 5-HT_1__A_ (Born et al., 2015; Albert et al., 2019). Moreover, quantitative results are also verified by hierarchical clustering. Hierarchical clustering shows that 370 differentially expressed proteins are separated into two obvious distinguishing branches, which indicates that the quantitative results are distinct and credible. Then, the function of differentially expressed proteins is analyzed by GO functional annotations and enrichment analysis. GO functional annotations find binding is the largest proportion of molecular function of differentially expressed proteins. GO enrichment analysis finds phosphorylation is the main biological process of differentially expressed proteins. These results indicate differentially expressed proteins which regulate phosphorylation by non-covalently binding may be the molecular targets of baicalin in hypothalamus. Next, protein-protein interaction analysis finds several high degree of nodes in neural network, such as 14-3-3 eta (Ywhah), 14-3-3 epsilon (Ywhae), 14-3-3 zeta (Ywhaz), NADPH-dependent 3-keto-steroid reductase Hsd3b4 (Hsd3b4), and nuclear pore complex protein Nup98-Nup96 (Nup98). High degree of nodes usually play a crucial part in protein-protein interaction network. In accordance with the results of GO, the function of above high degree nodes mainly focuses on GR signaling pathway by phosphoserine binding and phosphorylation. Previous studies show that GR is the crucial target of baicalin for antidepressant effects in olfactory bulb and hippocampus (Zhang et al., 2016; Gao et al., 2018), which indicates that GR is also the pivotal target of baicalin in hypothalamus for depression. Taken together, proteomics and systems biology data find that baicalin regulates some noteworthy proteins which are involved in GR signaling pathway, and indicate GR signaling pathway may be the regulatory mechanism of baicalin on negative feedback of HPA axis.

According to the results of proteomics and systems biology, we focus on the functions of GR in GR signaling pathway in hypothalamus. GR is one of the nuclear receptor subfamily members, which binds glucocorticoids and controls metabolism, development, and immune (Myers et al., 2014). More importantly, GR is also the control core for negative feedback of HPA axis in hypothalamus (Pariante and Lightman, 2008). Some studies show chronic stress decreases the expression of GR in hypothalamus (Cai et al., 2015). Therefore, we first assess the expression of total GR in hypothalamus. Whereas, the level of total GR is not affected by chronic CORT or baicalin. This result is in accordance with some studies, which chronic CORT do not change the expression of GR in hypothalamus (Wu et al., 2013). As we know, the function of GR is executed via GR transfers from the cytoplasm to the nucleus. So we next assess the level of GR in cytoplasm and nucleus. Interestingly, chronic CORT significantly decreases GR level in cytoplasm and increases GR level in nucleus, which indicates GR nuclear translocation is abnormality. This abnormal GR nuclear translocation may enhance the negative effects of GR and result in damaged negative feedback of HPA axis (Myers et al., 2014). After baicalin treatment, the distribution of GR in cytoplasm and nucleus are normalized. Noteworthy, fluoxetine normalizes the chronic CORT-induced increase in GR nuclear translocation and also does not affect total GR protein levels. Interestingly, this results are in line with the proteomics and systems biology analysis which baicalin can regulate differentially expressed proteins in GR signaling pathway. Moreover, many studies suggest that the phosphorylation status of the crucial serine residues on GR play the important role in regulating GR nuclear translocation (Guidotti et al., 2013). Proteomics and systems biology results also indicate that protein phosphorylation is the molecular targets of baicalin. Therefore, phosphorylation status of the crucial serine residues on GR in hypothalamus are assessed. Many studies showed that that phosphorylation at sites Ser203 and Ser211 facilitate nuclear translocation and increase the transcriptional activities of the receptor, while conversely, phosphorylation at site Ser226 inhibits nuclear translocation and decreases GR transcriptional activities (Anacker et al., 2013). Our data find that pSer203 and pSer211 level are remarkably increased in cytoplasm under chronic CORT treatment, which can enhance GR nuclear translocation (Anacker et al., 2013). This result also explains the reason why GR level in nucleus is increased. Baicalin can reduce pSer203 and pSer211 level in GR, and then recover this abnormal GR nuclear translocation. In accordance with this result, our previous study also found that baicalin could promote hippocampal neurogenesis by mediating GR phosphorylation (Zhang et al., 2016). Moreover, some studies have proposed that cAMP/PKA signaling pathway is involved in GR function (Anacker et al., 2011b). GR could interact with TrkB to promotes BDNF-triggered PLC-γ signaling pathway (Numakawa et al., 2009). However, definite downstream signals which can be affected by GR are still vague. Taken together, our results reveal baicalin normalizes GR nuclear translocation via reducing GR phosphorylation in hypothalamus, and then restores negative feedback of HPA axis.

## Conclusion

Our results demonstrate that baicalin remarkably improves chronic CORT-induced various depression-like behaviors and restores the negative feedback of HPA axis. Proteomics and systems biology indicate that the molecular mechanisms of baicalin involve normalizing GR nuclear translocation via regulating GR phosphorylation in hypothalamus. Our findings provide the new perspective on molecular targets of baicalin and will facilitate its clinical application.

## Data Availability

The raw data supporting the conclusions of this manuscript will be made available by the authors, without undue reservation, to any qualified researcher.

## Ethics Statement

This study was carried out in accordance with the principles of National Institutes of Health Guide for the Care and Use of Laboratory Animals (Publication No. 85-23). All efforts were made to minimize suffering.

## Author Contributions

KZ, JY, CW, YL, and HZ designed the study and drafted the manuscript. KZ, MH, and FW carried out the study. All authors read and approved the final manuscript.

## Conflict of Interest Statement

The authors declare that the research was conducted in the absence of any commercial or financial relationships that could be construed as a potential conflict of interest.
