# Supplementary material for: Revealing Antidepressant Mechanisms of Baicalin in Hypothalamus Through Systems Approaches in Corticosterone- Induced Depressed Mice
Source: Front Neurosci. 2019 Aug 8;13:834. doi: 10.3389/fnins.2019.00834 (PMC6694791; doi:10.3389/fnins.2019.00834)
Supplement: Supplementary file 1 [file Table_1.DOCX]

Table S1 Effects of baicalin on chronic CORT-induced differential proteins.

| NO. | Accession | Description | baicalin/model | | *P* value |
| --- | --- | --- | --- | --- | --- |
| 1 | Q8VEK3 | Heterogeneous nuclear ribonucleoprotein U | ↑ | 0.00006 | |
| 2 | S4R1K5 | Rho guanine nucleotide exchange factor 9 | ↑ | 0.00012 | |
| 3 | E0CYQ2 | NudC domain-containing protein 2 | ↑ | 0.00014 | |
| 4 | P48428 | Tubulin-specific chaperone A | ↑ | 0.00016 | |
| 5 | Q5SUH7 | Clathrin interactor 1 | ↑ | 0.00044 | |
| 6 | F8WI95 | Methylthioribose-1-phosphate isomerase (Fragment) | ↑ | 0.00102 | |
| 7 | Q6PDL0 | Cytoplasmic dynein 1 light intermediate chain 2 | ↑ | 0.00113 | |
| 8 | Q8R0A7 | Uncharacterized protein KIAA0513 | ↑ | 0.00185 | |
| 9 | Q8BUM1 | Putative uncharacterized protein | ↑ | 0.00213 | |
| 10 | Q9ERA0 | Alpha-globin transcription factor CP2 | ↑ | 0.00213 | |
| 11 | P62774 | Myotrophin | ↑ | 0.00218 | |
| 12 | Q3TCD4 | Enoyl-CoA delta isomerase 2, mitochondrial | ↑ | 0.00240 | |
| 13 | Q9CXR1 | Dehydrogenase/reductase SDR family member 7 | ↑ | 0.00275 | |
| 14 | Q91VR7 | Microtubule-associated proteins 1A/1B light chain 3A | ↑ | 0.00307 | |
| 15 | Z4YKM2 | CDGSH iron-sulfur domain-containing protein 3, mitochondrial | ↑ | 0.00307 | |
| 16 | P68510 | 14-3-3 protein eta | ↑ | 0.00370 | |
| 17 | Q9WTX2 | Interferon-inducible double-stranded RNA-dependent protein kinase activator A | ↑ | 0.00388 | |
| 18 | Q9QWW1 | Homer protein homolog 2 | ↑ | 0.00391 | |
| 19 | A0A0R4J1R7 | Pterin-4-alpha-carbinolamine dehydratase 2 | ↑ | 0.00422 | |
| 20 | Q8C000 | Putative uncharacterized protein | ↑ | 0.00449 | |
| 21 | Q3TVB4 | Putative uncharacterized protein (Fragment) | ↑ | 0.00459 | |
| 22 | Q9D7P6 | Iron-sulfur cluster assembly enzyme ISCU, mitochondrial | ↑ | 0.00481 | |
| 23 | Q3UGN9 | Signal transducing adapter molecule 1 | ↑ | 0.00500 | |
| 24 | F6SXM5 | Lupus La protein homolog (Fragment) | ↑ | 0.00542 | |
| 25 | Q8K2I1 | Protein farnesyltransferase subunit beta | ↑ | 0.00559 | |
| 26 | F8WHU9 | Zinc finger protein ZPR1 (Fragment) | ↑ | 0.00576 | |
| 27 | P28667 | MARCKS-related protein | ↑ | 0.00582 | |
| 28 | Q60865 | Caprin-1 | ↑ | 0.00592 | |
| 29 | Q3UQD2 | Putative uncharacterized protein (Fragment) | ↑ | 0.00605 | |
| 30 | Q99KR7 | Peptidyl-prolyl cis-trans isomerase F, mitochondrial | ↑ | 0.00608 | |
| 31 | Q9CZX8 | 40S ribosomal protein S19 | ↑ | 0.00619 | |
| 32 | H3BJ97 | Tubulointerstitial nephritis antigen-like | ↑ | 0.00648 | |
| 33 | Q3TSX8 | Putative uncharacterized protein | ↑ | 0.00661 | |
| 34 | P62869 | Transcription elongation factor B polypeptide 2 | ↑ | 0.00704 | |
| 35 | B2RY70 | 4933407C03Rik protein | ↑ | 0.00740 | |
| 36 | P11679 | Keratin, type II cytoskeletal 8 | ↑ | 0.00741 | |
| 37 | Q3UZN1 | Putative uncharacterized protein | ↑ | 0.00797 | |
| 38 | Q8BJZ7 | Putative uncharacterized protein | ↑ | 0.00844 | |
| 39 | Q62417 | Sorbin and SH3 domain-containing protein 1 | ↑ | 0.00860 | |
| 40 | Q05DV0 | Tyrosine-protein kinase | ↑ | 0.00868 | |
| 41 | Q9DBR7 | Protein phosphatase 1 regulatory subunit 12A | ↑ | 0.00954 | |
| 42 | Q3UGC7 | Eukaryotic translation initiation factor 3 subunit J-A | ↑ | 0.00965 | |
| 43 | A0A0A0MQM0 | Eukaryotic translation initiation factor 5A (Fragment) | ↑ | 0.01007 | |
| 44 | Q8VE52 | Opioid growth factor receptor-like protein 1 | ↑ | 0.01042 | |
| 45 | Q9D394 | Protein RUFY3 | ↑ | 0.01044 | |
| 46 | Q91XD7 | Cysteine-rich with EGF-like domain protein 1 | ↑ | 0.01088 | |
| 47 | A0A0R4J1P2 | Tropomyosin alpha-3 chain | ↑ | 0.01116 | |
| 48 | Q8JZQ1 | Translocase of inner mitochondrial membrane 23 homolog | ↑ | 0.01131 | |
| 49 | D0VYV6 | Erythrocyte protein band 4.1-like 3 isoform B | ↑ | 0.01131 | |
| 50 | Q9WVJ2 | 26S proteasome non-ATPase regulatory subunit 13 | ↑ | 0.01131 | |
| 51 | Q8BXP8 | Putative uncharacterized protein (Fragment) | ↑ | 0.01133 | |
| 52 | Q3TEF0 | Putative uncharacterized protein (Fragment) | ↑ | 0.01146 | |
| 53 | Q3TJ95 | Putative uncharacterized protein | ↑ | 0.01177 | |
| 54 | S4R1E5 | Glutathione peroxidase | ↑ | 0.01185 | |
| 55 | Q3TJN6 | Putative uncharacterized protein | ↑ | 0.01218 | |
| 56 | C6EQH3 | Succinyl-CoA ligase subunit beta | ↑ | 0.01255 | |
| 57 | G3UZI2 | Heterogeneous nuclear ribonucleoprotein Q | ↑ | 0.01558 | |
| 58 | Q8BGR6 | ADP-ribosylation factor-like protein 15 | ↑ | 0.01564 | |
| 59 | P11031 | Activated RNA polymerase II transcriptional coactivator p15 | ↑ | 0.01619 | |
| 60 | P55821 | Stathmin-2 | ↑ | 0.01659 | |
| 61 | Q8BVU5 | ADP-ribose pyrophosphatase, mitochondrial | ↑ | 0.01665 | |
| 62 | C9K0Y3 | AMPA-selective glutamate receptor 1 flip type | ↑ | 0.01836 | |
| 63 | A0A0G2JGP4 | GTPase NRas (Fragment) | ↑ | 0.01849 | |
| 64 | E9Q6I2 | Interleukin-1 receptor accessory protein | ↑ | 0.01859 | |
| 65 | Q3T9Y9 | Putative uncharacterized protein (Fragment) | ↑ | 0.01893 | |
| 66 | P11087 | Collagen alpha-1(I) chain | ↑ | 0.01943 | |
| 67 | E9Q9L1 | Sphingomyelin phosphodiesterase 4 | ↑ | 0.01966 | |
| 68 | Q3U7I9 | Putative uncharacterized protein | ↑ | 0.01992 | |
| 69 | Q9CR98 | Protein FAM136A | ↑ | 0.02058 | |
| 70 | P26041 | Moesin | ↑ | 0.02233 | |
| 71 | Q61081 | Hsp90 co-chaperone Cdc37 | ↑ | 0.02246 | |
| 72 | B2RSI3 | Poly [ADP-ribose] polymerase | ↑ | 0.02297 | |
| 73 | Q3U9U5 | Putative uncharacterized protein | ↑ | 0.02361 | |
| 74 | Q8R5C0 | Metaxin | ↑ | 0.02615 | |
| 75 | D3Z742 | MCG125361 | ↑ | 0.02678 | |
| 76 | Q8R0T3 | Wdr46 protein (Fragment) | ↑ | 0.02736 | |
| 77 | P32067 | Lupus La protein homolog | ↑ | 0.02748 | |
| 78 | O88952 | Protein lin-7 homolog C | ↑ | 0.02815 | |
| 79 | P40240 | CD9 antigen | ↑ | 0.02840 | |
| 80 | E9PUD2 | Dynamin-1-like protein | ↑ | 0.02884 | |
| 81 | B2KGA7 | Protein archease | ↑ | 0.02892 | |
| 82 | Q3TZM9 | GDP-Man:Man(3)GlcNAc(2)-PP-Dol alpha-1,2-mannosyltransferase | ↑ | 0.02941 | |
| 83 | H3BL13 | N-acylethanolamine-hydrolyzing acid amidase (Fragment) | ↑ | 0.03029 | |
| 84 | Q8BMK4 | Cytoskeleton-associated protein 4 | ↑ | 0.03040 | |
| 85 | Q924W7 | Suppression of tumorigenicity 5 protein | ↑ | 0.03099 | |
| 86 | P40237 | CD82 antigen | ↑ | 0.03106 | |
| 87 | F8WHT2 | Protein phosphatase Slingshot homolog 1 | ↑ | 0.03263 | |
| 88 | Q3U671 | Putative uncharacterized protein | ↑ | 0.03265 | |
| 89 | Q91V77 | Protein S100 | ↑ | 0.03337 | |
| 90 | O08797 | Protein Serpinb9 | ↑ | 0.03515 | |
| 91 | Q8R5J9 | PRA1 family protein 3 | ↑ | 0.03547 | |
| 92 | Q9Z204 | Heterogeneous nuclear ribonucleoproteins C1/C2 | ↑ | 0.03549 | |
| 93 | Q59J78 | Mimitin, mitochondrial | ↑ | 0.03625 | |
| 94 | Q9R1Z7 | 6-pyruvoyl tetrahydrobiopterin synthase | ↑ | 0.03665 | |
| 95 | A1L146 | Lzts1 protein (Fragment) | ↑ | 0.03697 | |
| 96 | Q99N92 | 39S ribosomal protein L27, mitochondrial | ↑ | 0.03754 | |
| 97 | Q8K2Y7 | 39S ribosomal protein L47, mitochondrial | ↑ | 0.03784 | |
| 98 | Q8K0T4 | Katanin p60 ATPase-containing subunit A-like 1 | ↑ | 0.03829 | |
| 99 | F6QKK2 | ADP-ribosylation factor-like protein 8A (Fragment) | ↑ | 0.03852 | |
| 100 | Q99MR1 | PERQ amino acid-rich with GYF domain-containing protein 1 | ↑ | 0.04022 | |
| 101 | F8VQC1 | Signal recognition particle subunit SRP72 | ↑ | 0.04073 | |
| 102 | P46978 | Dolichyl-diphosphooligosaccharide--protein glycosyltransferase subunit STT3A | ↑ | 0.04155 | |
| 103 | D3YXK2 | Scaffold attachment factor B1 | ↑ | 0.04158 | |
| 104 | P17047 | Lysosome-associated membrane glycoprotein 2 | ↑ | 0.04162 | |
| 105 | F8WIT2 | Annexin | ↑ | 0.04174 | |
| 106 | Q91X78 | Erlin-1 | ↑ | 0.04196 | |
| 107 | Q9JKD3 | Secretory carrier-associated membrane protein 5 | ↑ | 0.04329 | |
| 108 | F8WIS9 | Calcium/calmodulin-dependent protein kinase type II subunit alpha | ↑ | 0.04408 | |
| 109 | Q9CY45 | Protein-lysine N-methyltransferase N6amt2 | ↑ | 0.04438 | |
| 110 | Q50H33 | BTB/POZ domain-containing protein KCTD8 | ↑ | 0.04462 | |
| 111 | Q6P208 | Regulator of G-protein signaling 17 | ↑ | 0.04469 | |
| 112 | Q9D1R9 | 60S ribosomal protein L34 | ↑ | 0.04652 | |
| 113 | A0A0R4J1N9 | Transcription factor A, mitochondrial | ↑ | 0.04735 | |
| 114 | Q45VK7 | Cytoplasmic dynein 2 heavy chain 1 | ↑ | 0.04941 | |
| 115 | Q3UZP4 | Small VCP/p97-interacting protein | ↓ | 0.00003 | |
| 116 | Q8R3U8 | Mapkapk2 protein (Fragment) | ↓ | 0.00007 | |
| 117 | A0A087WP24 | Alpha/beta hydrolase domain-containing protein 14B (Fragment) | ↓ | 0.00010 | |
| 118 | Q8C660 | RIKEN cDNA 1810008A18 | ↓ | 0.00011 | |
| 119 | Q3TB82 | Pleckstrin homology domain-containing family F member 1 | ↓ | 0.00019 | |
| 120 | Q6PFD9 | Nuclear pore complex protein Nup98-Nup96 | ↓ | 0.00022 | |
| 121 | A2AR81 | T-box brain protein 1 | ↓ | 0.00026 | |
| 122 | D3Z1C5 | LIM domain-binding protein 1 | ↓ | 0.00028 | |
| 123 | Q8BL03 | Mitochondrial basic amino acids transporter | ↓ | 0.00033 | |
| 124 | Q3UGZ2 | Putative uncharacterized protein | ↓ | 0.00043 | |
| 125 | A0A0G2JFT8 | Protein RUFY3 | ↓ | 0.00056 | |
| 126 | P70165 | Collagen type IV alpha5 chain (Fragment) | ↓ | 0.00079 | |
| 127 | Q4VA53 | Sister chromatid cohesion protein PDS5 homolog B | ↓ | 0.00082 | |
| 128 | Q3UHE1 | Membrane-associated phosphatidylinositol transfer protein 3 | ↓ | 0.00090 | |
| 129 | Q64520 | Guanylate kinase | ↓ | 0.00093 | |
| 130 | Q8C3X2 | Coiled-coil domain-containing protein 90B, mitochondrial | ↓ | 0.00097 | |
| 131 | C0IXK2 | Interferon regulatory factor 3 transcript variant a | ↓ | 0.00099 | |
| 132 | Q8K238 | Rpp30 protein (Fragment) | ↓ | 0.00100 | |
| 133 | A0A0N4SVL0 | Eukaryotic translation initiation factor 4 gamma 3 | ↓ | 0.00100 | |
| 134 | Q8BGT5 | Alanine aminotransferase 2 | ↓ | 0.00100 | |
| 135 | Q8VI51 | VPS10 domain-containing receptor SorCS3 | ↓ | 0.00102 | |
| 136 | Q8K2U5 | Sec63 protein (Fragment) | ↓ | 0.00103 | |
| 137 | Q6PCZ4 | Melanoma-associated antigen E1 | ↓ | 0.00103 | |
| 138 | D3Z5P0 | Serine/threonine-protein kinase BRSK1 | ↓ | 0.00107 | |
| 139 | Q9CXS4 | Centromere protein V | ↓ | 0.00112 | |
| 140 | A6H650 | MCG133012 | ↓ | 0.00127 | |
| 141 | A2AWT6 | Nucleolar transcription factor 1 | ↓ | 0.00136 | |
| 142 | F7CD65 | Epsin-2 (Fragment) | ↓ | 0.00142 | |
| 143 | A0A0U1RP81 | MICOS complex subunit Mic60 | ↓ | 0.00145 | |
| 144 | Q8CH18 | Cell division cycle and apoptosis regulator protein 1 | ↓ | 0.00149 | |
| 145 | Q8BG92 | Clavesin-2 | ↓ | 0.00156 | |
| 146 | D3YVS7 | Cysteine-rich and transmembrane domain-containing protein 1 (Fragment) | ↓ | 0.00156 | |
| 147 | A0A0R4J050 | Aminoacylase-1 | ↓ | 0.00168 | |
| 148 | Q80XU8 | Leucine-rich repeat and fibronectin type-III domain-containing protein 4 | ↓ | 0.00174 | |
| 149 | E9QPQ8 | 39S ribosomal protein L48, mitochondrial | ↓ | 0.00183 | |
| 150 | Q8BTR5 | Dual specificity phosphatase 28 | ↓ | 0.00186 | |
| 151 | F8WHU8 | SRA stem-loop-interacting RNA-binding protein, mitochondrial (Fragment) | ↓ | 0.00196 | |
| 152 | Q91W61 | F-box/LRR-repeat protein 15 | ↓ | 0.00197 | |
| 153 | A0A0A0MQI4 | Disabled homolog 1 (Fragment) | ↓ | 0.00198 | |
| 154 | Q8BVB0 | Non-specific serine/threonine protein kinase | ↓ | 0.00247 | |
| 155 | Q99NE5 | Regulating synaptic membrane exocytosis protein 1 | ↓ | 0.00255 | |
| 156 | Q9D6Y7 | Mitochondrial peptide methionine sulfoxide reductase | ↓ | 0.00261 | |
| 157 | P34884 | Macrophage migration inhibitory factor | ↓ | 0.00272 | |
| 158 | Q80TA9 | Ectopic P granules protein 5 homolog | ↓ | 0.00277 | |
| 159 | Q8CBU3 | Putative uncharacterized protein | ↓ | 0.00282 | |
| 160 | Q8VHY0 | Chondroitin sulfate proteoglycan 4 | ↓ | 0.00298 | |
| 161 | Q3V3S8 | Putative uncharacterized protein | ↓ | 0.00301 | |
| 162 | Q91YZ2 | C-terminal binding protein 2 | ↓ | 0.00314 | |
| 163 | Q3UEW8 | Putative uncharacterized protein (Fragment) | ↓ | 0.00321 | |
| 164 | Q3UGS0 | Putative uncharacterized protein | ↓ | 0.00343 | |
| 165 | Q8R191 | Synaptogyrin-3 | ↓ | 0.00345 | |
| 166 | Q9CQ00 | Transmembrane protein 261 | ↓ | 0.00347 | |
| 167 | P63087 | Serine/threonine-protein phosphatase PP1-gamma catalytic subunit | ↓ | 0.00347 | |
| 168 | Q6P5E8 | Diacylglycerol kinase theta | ↓ | 0.00350 | |
| 169 | Q5SVF8 | THO complex subunit 5 homolog (Fragment) | ↓ | 0.00361 | |
| 170 | Q7TMW6 | Cytosolic Fe-S cluster assembly factor NARFL | ↓ | 0.00370 | |
| 171 | P16283 | Anion exchange protein 3 | ↓ | 0.00408 | |
| 172 | O35972 | 39S ribosomal protein L23, mitochondrial | ↓ | 0.00424 | |
| 173 | Q8C8H5 | Putative uncharacterized protein | ↓ | 0.00431 | |
| 174 | Q9D8S9 | BolA-like protein 1 | ↓ | 0.00444 | |
| 175 | P08122 | Collagen alpha-2(IV) chain | ↓ | 0.00456 | |
| 176 | Q99KR3 | Beta-lactamase-like protein 2 | ↓ | 0.00457 | |
| 177 | F7BHW1 | Cadherin EGF LAG seven-pass G-type receptor 2 (Fragment) | ↓ | 0.00488 | |
| 178 | Q6ZWX6 | Eukaryotic translation initiation factor 2 subunit 1 | ↓ | 0.00509 | |
| 179 | A4GZ26 | ARF6 guanine nucleotide exchange factor IQArfGEF | ↓ | 0.00519 | |
| 180 | P70415 | TR2L (Fragment) | ↓ | 0.00519 | |
| 181 | F6UK66 | Coiled-coil domain-containing protein 50 (Fragment) | ↓ | 0.00563 | |
| 182 | D3YZV2 | Potassium voltage-gated channel subfamily C member 3 | ↓ | 0.00589 | |
| 183 | Q8BI08 | Protein MAL2 | ↓ | 0.00595 | |
| 184 | Q9QZI8 | Serine incorporator 1 | ↓ | 0.00595 | |
| 185 | A0A0J9YVG0 | Protein phosphatase 1G | ↓ | 0.00644 | |
| 186 | Q9D504 | Ankyrin repeat domain-containing protein 7 | ↓ | 0.00669 | |
| 187 | Q3THK3 | General transcription factor IIF subunit 1 | ↓ | 0.00686 | |
| 188 | E9QAJ9 | Rho GTPase-activating protein 17 | ↓ | 0.00698 | |
| 189 | O08908 | Phosphatidylinositol 3-kinase regulatory subunit beta | ↓ | 0.00700 | |
| 190 | O08915 | AH receptor-interacting protein | ↓ | 0.00701 | |
| 191 | F7C106 | Cytochrome c oxidase subunit 5B, mitochondrial (Fragment) | ↓ | 0.00705 | |
| 192 | F6SPK0 | Ubiquitin-conjugating enzyme E2 J1 (Fragment) | ↓ | 0.00710 | |
| 193 | Q9ESN4 | Complement C1q-like protein 3 | ↓ | 0.00718 | |
| 194 | P97813 | Phospholipase D2 | ↓ | 0.00726 | |
| 195 | Q3TRC8 | Putative uncharacterized protein | ↓ | 0.00728 | |
| 196 | A7YL62 | Apolipoprotein A-II | ↓ | 0.00740 | |
| 197 | O35619 | Vesicle associated membrane protein 2 | ↓ | 0.00745 | |
| 198 | Q62084 | Protein phosphatase 1 regulatory subunit 14B | ↓ | 0.00752 | |
| 199 | Q68G78 | Csda protein | ↓ | 0.00776 | |
| 200 | Q6ZWM4 | U6 snRNA-associated Sm-like protein LSm8 | ↓ | 0.00787 | |
| 201 | Q3UZT2 | Putative uncharacterized protein | ↓ | 0.00808 | |
| 202 | Q9DBD5 | Proline-, glutamic acid- and leucine-rich protein 1 | ↓ | 0.00810 | |
| 203 | D3Z6S7 | Immunoglobulin superfamily member 11 | ↓ | 0.00828 | |
| 204 | B2RX09 | Nrxn2 protein | ↓ | 0.00830 | |
| 205 | Q8VDS8 | Syntaxin-18 | ↓ | 0.00872 | |
| 206 | Q3UTE4 | Putative uncharacterized protein (Fragment) | ↓ | 0.00931 | |
| 207 | Q91WU5 | Arsenite methyltransferase | ↓ | 0.00931 | |
| 208 | F6Z0Z5 | GTP-binding protein REM 2 (Fragment) | ↓ | 0.00951 | |
| 209 | Q2VPC9 | Prkag1 protein (Fragment) | ↓ | 0.00955 | |
| 210 | Q3UXM3 | Putative uncharacterized protein | ↓ | 0.00957 | |
| 211 | D6RFA8 | Acyltransferase like 2 | ↓ | 0.00970 | |
| 212 | E9Q0W8 | Small nuclear ribonucleoprotein E | ↓ | 0.00992 | |
| 213 | F6RXH3 | Protein phosphatase 1 regulatory subunit 3F (Fragment) | ↓ | 0.00992 | |
| 214 | Q9DCM0 | Persulfide dioxygenase ETHE1, mitochondrial | ↓ | 0.01006 | |
| 215 | Q8VCQ8 | Caldesmon 1 | ↓ | 0.01009 | |
| 216 | P49817 | Caveolin-1 | ↓ | 0.01022 | |
| 217 | Q8BWZ3 | N-alpha-acetyltransferase 25, NatB auxiliary subunit | ↓ | 0.01026 | |
| 218 | Q3TDU5 | Milk fat globule-EGF factor 8 protein | ↓ | 0.01050 | |
| 219 | A2AFI8 | RalBP1-associated Eps domain-containing protein 2 | ↓ | 0.01063 | |
| 220 | Q5DTP6 | MKIAA4076 protein (Fragment) | ↓ | 0.01070 | |
| 221 | Q9CVT6 | Putative uncharacterized protein (Fragment) | ↓ | 0.01078 | |
| 222 | Q9Z0L0 | Trophoblast glycoprotein | ↓ | 0.01099 | |
| 223 | Q3UK08 | Putative uncharacterized protein | ↓ | 0.01103 | |
| 224 | Q8R3U1 | HRAS-like suppressor 3 | ↓ | 0.01137 | |
| 225 | E9QM90 | Protein 2310035C23Rik | ↓ | 0.01140 | |
| 226 | Q03157 | Amyloid-like protein 1 | ↓ | 0.01157 | |
| 227 | Q0GA42 | Metal transporter CNNM1 | ↓ | 0.01177 | |
| 228 | Q91XC9 | Peroxisomal membrane protein PEX16 | ↓ | 0.01260 | |
| 229 | E9PUL6 | Sodium/potassium/calcium exchanger 4 | ↓ | 0.01283 | |
| 230 | Q9D6W8 | Uncharacterized protein C17orf59 homolog | ↓ | 0.01300 | |
| 231 | P60840 | Alpha-endosulfine | ↓ | 0.01309 | |
| 232 | Q91Z92 | Beta-1,3-galactosyltransferase 6 | ↓ | 0.01338 | |
| 233 | D3YXY5 | DET1- and DDB1-associated protein 1 | ↓ | 0.01354 | |
| 234 | V9GX85 | Netrin-G2 | ↓ | 0.01356 | |
| 235 | Q0VGU5 | Vitamin K epoxide reductase complex subunit 1-like protein 1 | ↓ | 0.01369 | |
| 236 | A0A0R4J0T0 | Iron-sulfur cluster co-chaperone protein HscB, mitochondrial | ↓ | 0.01370 | |
| 237 | Q05CJ8 | Golga1 protein (Fragment) | ↓ | 0.01372 | |
| 238 | A0A0U1RP47 | E3 ubiquitin-protein ligase CBL | ↓ | 0.01395 | |
| 239 | Q3U912 | Putative uncharacterized protein | ↓ | 0.01398 | |
| 240 | Q9D1L9 | Ragulator complex protein LAMTOR5 | ↓ | 0.01434 | |
| 241 | Q5I0W5 | Sdf2 protein | ↓ | 0.01515 | |
| 242 | Q3T992 | Putative uncharacterized protein | ↓ | 0.01536 | |
| 243 | Q9JJF3 | Bifunctional lysine-specific demethylase and histidyl-hydroxylase NO66 | ↓ | 0.01541 | |
| 244 | Q64264 | 5-hydroxytryptamine receptor 1A | ↓ | 0.01593 | |
| 245 | A2AV25 | Fibrinogen C domain-containing protein 1 | ↓ | 0.01607 | |
| 246 | Q80YD1 | ATP-dependent RNA helicase SUPV3L1, mitochondrial | ↓ | 0.01621 | |
| 247 | Q99JP0 | Mitogen-activated protein kinase kinase kinase kinase 3 | ↓ | 0.01627 | |
| 248 | Q921H8 | 3-ketoacyl-CoA thiolase A, peroxisomal | ↓ | 0.01655 | |
| 249 | D3YW10 | Ubiquitin-conjugating enzyme E2 E2 (Fragment) | ↓ | 0.01662 | |
| 250 | O54724 | Polymerase I and transcript release factor | ↓ | 0.01672 | |
| 251 | Q8R554 | OTU domain-containing protein 7A | ↓ | 0.01687 | |
| 252 | A8Y5C3 | PDZ and LIM domain protein 4 (Fragment) | ↓ | 0.01720 | |
| 253 | Q61923 | Potassium voltage-gated channel subfamily A member 6 | ↓ | 0.01723 | |
| 254 | Q80WT5 | Aftiphilin | ↓ | 0.01727 | |
| 255 | Q9JIG8 | PRA1 family protein 2 | ↓ | 0.01808 | |
| 256 | Q9WTI7 | Unconventional myosin-Ic | ↓ | 0.01824 | |
| 257 | Q8BQP9 | Regulator of G-protein signaling 7-binding protein | ↓ | 0.01843 | |
| 258 | A8IP73 | NAA-3 protein | ↓ | 0.01917 | |
| 259 | Q6PDG0 | Nup205 protein (Fragment) | ↓ | 0.01933 | |
| 260 | A2VCP7 | Psmf1 protein (Fragment) | ↓ | 0.01940 | |
| 261 | P58069 | Ras GTPase-activating protein 2 | ↓ | 0.01983 | |
| 262 | O55135 | Eukaryotic translation initiation factor 6 | ↓ | 0.01987 | |
| 263 | Q3TXY2 | Putative uncharacterized protein | ↓ | 0.01987 | |
| 264 | A0A0N4SWD7 | Protein Plekha5 (Fragment) | ↓ | 0.01991 | |
| 265 | Q9CSV9 | Putative uncharacterized protein (Fragment) | ↓ | 0.02015 | |
| 266 | A2AJ26 | ATP-binding cassette sub-family A member 2 | ↓ | 0.02037 | |
| 267 | Q5QNQ6 | Oxysterol-binding protein 2 | ↓ | 0.02062 | |
| 268 | Q4QQN0 | Acap3 protein | ↓ | 0.02071 | |
| 269 | Q3TQP0 | Protein Gm10767 | ↓ | 0.02074 | |
| 270 | Q61823 | Programmed cell death protein 4 | ↓ | 0.02074 | |
| 271 | Q3UUJ4 | STE20-related kinase adapter protein alpha | ↓ | 0.02082 | |
| 272 | Q8CJ61 | CKLF-like MARVEL transmembrane domain-containing protein 4 | ↓ | 0.02104 | |
| 273 | Q3UDE2 | Tubulin--tyrosine ligase-like protein 12 | ↓ | 0.02104 | |
| 274 | Q8CE96 | tRNA (adenine(58)-N(1))-methyltransferase non-catalytic subunit TRM6 | ↓ | 0.02107 | |
| 275 | Q6PD24 | Ankyrin repeat domain-containing protein 13D | ↓ | 0.02216 | |
| 276 | H3BLC3 | Neuralized-like protein 4 (Fragment) | ↓ | 0.02256 | |
| 277 | Q8CH25 | SAFB-like transcription modulator | ↓ | 0.02323 | |
| 278 | F6U3S2 | Major facilitator superfamily domain-containing protein 6 (Fragment) | ↓ | 0.02344 | |
| 279 | J3QNY1 | Protein Gm9242 | ↓ | 0.02346 | |
| 280 | Q8R480 | Nuclear pore complex protein Nup85 | ↓ | 0.02354 | |
| 281 | Q3TDE4 | Putative uncharacterized protein (Fragment) | ↓ | 0.02372 | |
| 282 | Q3TZJ2 | Putative uncharacterized protein | ↓ | 0.02405 | |
| 283 | Q8BMD8 | Calcium-binding mitochondrial carrier protein SCaMC-1 | ↓ | 0.02422 | |
| 284 | Q8BR92 | Paralemmin-2 | ↓ | 0.02455 | |
| 285 | B8QI36 | Liprin-alpha 4 | ↓ | 0.02490 | |
| 286 | Q3TJI8 | Corticosteroid 11-beta-dehydrogenase isozyme 1 | ↓ | 0.02522 | |
| 287 | Q8BWR2 | PITH domain-containing protein 1 | ↓ | 0.02541 | |
| 288 | E9Q741 | Chloride transport protein 6 | ↓ | 0.02577 | |
| 289 | P62311 | U6 snRNA-associated Sm-like protein LSm3 | ↓ | 0.02583 | |
| 290 | A0A087WRN1 | Bcl-2-associated transcription factor 1 (Fragment) | ↓ | 0.02601 | |
| 291 | A3KFX0 | Cytosolic 5'-nucleotidase 1A | ↓ | 0.02619 | |
| 292 | Q3SXD3 | HD domain-containing protein 2 | ↓ | 0.02621 | |
| 293 | B9EHT4 | CAP-Gly domain-containing linker protein 3 | ↓ | 0.02621 | |
| 294 | Q3U487 | E3 ubiquitin-protein ligase HECTD3 | ↓ | 0.02674 | |
| 295 | E9PUZ5 | PRKCA-binding protein | ↓ | 0.02696 | |
| 296 | Q14BB9 | MAP6 domain-containing protein 1 | ↓ | 0.02700 | |
| 297 | Q0QEW9 | Ribosomal protein L18 (Fragment) | ↓ | 0.02707 | |
| 298 | Q02105 | Complement C1q subcomponent subunit C | ↓ | 0.02763 | |
| 299 | Q8VED9 | Galectin-related protein | ↓ | 0.02770 | |
| 300 | Q8BM13 | Noelin-2 | ↓ | 0.02813 | |
| 301 | D0EX61 | Muscleblind-like 2 isoform 3 | ↓ | 0.02832 | |
| 302 | G3X9D3 | Podocalyxin-like 2 | ↓ | 0.02849 | |
| 303 | E9PVU9 | Bis(5'-adenosyl)-triphosphatase (Fragment) | ↓ | 0.02861 | |
| 304 | Q8BHE3 | Caytaxin | ↓ | 0.02865 | |
| 305 | B2RXW8 | Ppfia1 protein | ↓ | 0.02877 | |
| 306 | Q3TIR3 | Synembryn-A | ↓ | 0.02913 | |
| 307 | D3Z286 | Protein Tmppe | ↓ | 0.02916 | |
| 308 | Q3U2A8 | Valine--tRNA ligase, mitochondrial | ↓ | 0.02948 | |
| 309 | Q3UXD8 | Ribosomal protein S6 kinase | ↓ | 0.02976 | |
| 310 | P70202 | Latexin | ↓ | 0.03043 | |
| 311 | Q8BYK6 | YTH domain-containing family protein 3 | ↓ | 0.03105 | |
| 312 | Q3V231 | Putative uncharacterized protein | ↓ | 0.03108 | |
| 313 | P61600 | N-alpha-acetyltransferase 20 | ↓ | 0.03126 | |
| 314 | Q8C1Y8 | Vacuolar fusion protein CCZ1 homolog | ↓ | 0.03149 | |
| 315 | Q8C5P7 | Testis development-related protein | ↓ | 0.03163 | |
| 316 | A3KMF2 | Mkl2 protein (Fragment) | ↓ | 0.03191 | |
| 317 | A0A023J5Y9 | ATP synthase protein 8 | ↓ | 0.03199 | |
| 318 | Q9CR00 | 26S proteasome non-ATPase regulatory subunit 9 | ↓ | 0.03227 | |
| 319 | Q80VM5 | Dipeptidyl aminopeptidase-like protein 6 | ↓ | 0.03256 | |
| 320 | Q9QZ08 | N-acetyl-D-glucosamine kinase | ↓ | 0.03289 | |
| 321 | Q80Y17 | Lethal(2) giant larvae protein homolog 1 | ↓ | 0.03295 | |
| 322 | E9PYH3 | Ethanolamine-phosphate phospho-lyase | ↓ | 0.03305 | |
| 323 | Q91W53 | Golgin subfamily A member 7 | ↓ | 0.03344 | |
| 324 | Q6ZQ29 | Serine/threonine-protein kinase TAO2 | ↓ | 0.03398 | |
| 325 | Q3U8Y1 | 28S ribosomal protein S11, mitochondrial | ↓ | 0.03441 | |
| 326 | A0A0J9YUR7 | Ubiquitin-conjugating enzyme E2 A | ↓ | 0.03467 | |
| 327 | A0A0R4J1T3 | Serine/threonine-protein kinase TAO3 (Fragment) | ↓ | 0.03477 | |
| 328 | Q921L3 | Transmembrane and coiled-coil domain-containing protein 1 | ↓ | 0.03536 | |
| 329 | Q6VNS1 | NT-3 growth factor receptor | ↓ | 0.03575 | |
| 330 | Q2HJ05 | Gap junction protein | ↓ | 0.03579 | |
| 331 | E9Q8K8 | Zinc finger CCCH domain-containing protein 4 | ↓ | 0.03580 | |
| 332 | O55003 | BCL2/adenovirus E1B 19 kDa protein-interacting protein 3 | ↓ | 0.03585 | |
| 333 | Q9D0G0 | 28S ribosomal protein S30, mitochondrial | ↓ | 0.03594 | |
| 334 | H3BJT9 | 39S ribosomal protein L17, mitochondrial (Fragment) | ↓ | 0.03616 | |
| 335 | Q9D2R0 | Acetoacetyl-CoA synthetase | ↓ | 0.03626 | |
| 336 | E9Q9C5 | V-type proton ATPase 16 kDa proteolipid subunit (Fragment) | ↓ | 0.03638 | |
| 337 | Q9CZX0 | Elongator complex protein 3 | ↓ | 0.03657 | |
| 338 | F6ZDS4 | Nucleoprotein TPR | ↓ | 0.03724 | |
| 339 | D6RET7 | GRAM domain-containing protein 4 | ↓ | 0.03739 | |
| 340 | Q8C3Q5 | Protein shisa-7 | ↓ | 0.03771 | |
| 341 | P54830 | Tyrosine-protein phosphatase non-receptor type 5 | ↓ | 0.03903 | |
| 342 | Q8C6M7 | Putative uncharacterized protein | ↓ | 0.04020 | |
| 343 | Q8BLY3 | Leucine-rich repeat and fibronectin type-III domain-containing protein 3 | ↓ | 0.04024 | |
| 344 | A2AHJ7 | Diacylglycerol kinase | ↓ | 0.04041 | |
| 345 | A0A0R4J0W7 | Serine/threonine-protein kinase LMTK3 | ↓ | 0.04061 | |
| 346 | Q8C4X2 | Casein kinase I isoform gamma-3 | ↓ | 0.04192 | |
| 347 | Q99LX5 | Multiple myeloma tumor-associated protein 2 homolog | ↓ | 0.04220 | |
| 348 | V9GXA7 | Protein Gm15294 | ↓ | 0.04226 | |
| 349 | Q3UGM4 | Anion exchange protein (Fragment) | ↓ | 0.04255 | |
| 350 | P46414 | Cyclin-dependent kinase inhibitor 1B | ↓ | 0.04261 | |
| 351 | Q8CAK1 | Putative transferase CAF17 homolog, mitochondrial | ↓ | 0.04274 | |
| 352 | Q7TQE2 | Zyx protein | ↓ | 0.04310 | |
| 353 | Z4YK42 | NAD kinase 2, mitochondrial | ↓ | 0.04322 | |
| 354 | Q9WUN2 | Serine/threonine-protein kinase TBK1 | ↓ | 0.04343 | |
| 355 | A0A0N4SV10 | Ethanolamine kinase 1 | ↓ | 0.04351 | |
| 356 | Q3ZB59 | GRB2-associated-binding protein 2 | ↓ | 0.04399 | |
| 357 | Q99KG3 | RNA-binding protein 10 | ↓ | 0.04424 | |
| 358 | Q5NCB5 | Immunity-related GTPase family M protein 1 (Fragment) | ↓ | 0.04458 | |
| 359 | Q9JMF0 | mRNA, clone:2-63 (Fragment) | ↓ | 0.04593 | |
| 360 | O55137 | Acyl-coenzyme A thioesterase 1 | ↓ | 0.04688 | |
| 361 | Q5DTK7 | MKIAA4158 protein (Fragment) | ↓ | 0.04744 | |
| 362 | Q8K2V6 | Importin-11 | ↓ | 0.04755 | |
| 363 | Q8BH50 | Uncharacterized protein C18orf25 homolog | ↓ | 0.04768 | |
| 364 | Q921T2 | Torsin-1A-interacting protein 1 | ↓ | 0.04854 | |
| 365 | Q9D6K5 | Synaptojanin-2-binding protein | ↓ | 0.04855 | |
| 366 | P60041 | Somatostatin | ↓ | 0.04858 | |
| 367 | Q6PAV2 | Probable E3 ubiquitin-protein ligase HERC4 | ↓ | 0.04877 | |
| 368 | Q6ZPP3 | MKIAA1507 protein (Fragment) | ↓ | 0.04946 | |
| 369 | Q8BPP6 | Putative uncharacterized protein | ↓ | 0.04955 | |
| 370 | O35449 | Proline-rich transmembrane protein 1 | ↓ | 0.04978 | |

Table S2 The name and biological process of high degree nodes under baicalin treatment in CORT-induced mice.

| Protein gene | Name | Molecular function | Biological process |
| --- | --- | --- | --- |
| Ywhae | 14-3-3 protein epsilon | enzyme binding  phosphoprotein binding | hippocampus development  negative regulation of protein dephosphorylation |
| Ywhaz | 14-3-3 protein zeta/delta | [protein kinase binding](http://www.ebi.ac.uk/QuickGO/GTerm?id=GO:0019901)  [transcription factor binding](http://www.ebi.ac.uk/QuickGO/GTerm?id=GO:0008134) | [regulation of cell death](http://www.ebi.ac.uk/QuickGO/GTerm?id=GO:0010941)  regulation of synapse maturation |
| Hsd3b4 | 3 beta-hydroxysteroid dehydrogenase type 4 | oxidoreductase activity  steroid binding | response to corticosterone  [steroid biosynthetic process](http://www.ebi.ac.uk/QuickGO/GTerm?id=GO:0006694) |
| Pgls | 6-phosphogluconolactonase | [monosaccharide binding](http://www.ebi.ac.uk/QuickGO/GTerm?id=GO:0048029) | [carbohydrate metabolic process](http://www.ebi.ac.uk/QuickGO/GTerm?id=GO:0005975) |
| Hsd3b7 | 3 beta-hydroxysteroid dehydrogenase type 7 | oxidoreductase activity | regulation of cell growth  [steroid biosynthetic process](http://www.ebi.ac.uk/QuickGO/GTerm?id=GO:0006694) |
| Lactb2 | Beta-lactamase-like protein 2 | endoribonuclease activity | RNA phosphodiester bond hydrolysis, endonucleolytic |
| Ywhah | 14-3-3 protein eta | [glucocorticoid receptor binding](http://www.ebi.ac.uk/QuickGO/GTerm?id=GO:0035259) | [glucocorticoid catabolic process](http://www.ebi.ac.uk/QuickGO/GTerm?id=GO:0006713)  [glucocorticoid receptor signaling pathway](http://www.ebi.ac.uk/QuickGO/GTerm?id=GO:0042921) |
| Hmga2 | High mobility group protein HMGI-C | [cAMP response element binding](http://www.ebi.ac.uk/QuickGO/GTerm?id=GO:0035497) | [adrenal gland development](http://www.ebi.ac.uk/QuickGO/GTerm?id=GO:0030325)  [cell proliferation](http://www.ebi.ac.uk/QuickGO/GTerm?id=GO:0008283) |
| Ywhab | 14-3-3 protein beta/alpha | [phosphoserine binding](http://www.ebi.ac.uk/QuickGO/GTerm?id=GO:0050815) | [negative regulation of protein dephosphorylation](http://www.ebi.ac.uk/QuickGO/GTerm?id=GO:0035308) |
| Ppp1cc | Serine/threonine-protein phosphatase PP1-gamma catalytic subunit | protein phosphatase 1 binding  protein phosphatase binding | protein serine/threonine phosphatase activity |
